# Skipping ahead: A circuit for representing the past, present, and future

**DOI:** 10.7554/eLife.68795

**Published:** 2021-10-14

**Authors:** Jennifer C Robinson, Mark P Brandon

**Affiliations:** 1 Department of Psychological and Brain Sciences, Rajen Kilachand Center for Integrated Life Sciences and Engineering, Boston University Boston United States; 2 Department of Psychiatry, Douglas Hospital Research Centre, McGill University Montreal Canada; University of Texas at Austin United States; University of Texas at Austin United States

**Keywords:** hippocampus, prefrontal cortex, cycle skipping, memory, theta sequences

## Abstract

Envisioning the future is intuitively linked to our ability to remember the past. Within the memory system, substantial work has demonstrated the involvement of the prefrontal cortex and the hippocampus in representing the past and present. Recent data shows that both the prefrontal cortex and the hippocampus encode future trajectories, which are segregated in time by alternating cycles of the theta rhythm. Here, we discuss how information is temporally organized by these brain regions supported by the medial septum, nucleus reuniens, and parahippocampal regions. Finally, we highlight a brain circuit that we predict is essential for the temporal segregation of future scenarios.

## Introduction

Our ability to imagine the future is nested in our capacity to remember the past. At their most basic description, memory and imagination are both cognitive processes in which mental constructs can be formed in the absence of directly relevant sensory information. It is not surprising that both processes draw on input from similar brain regions, as evidenced by human neuroimaging studies reporting that frontal cortices and the hippocampus are activated during both memory retrieval and planning or imagination. Individuals with memory impairments report difficulty in planning and imagination (Hassabis et al., 2007) and when asked to imagine future possible scenarios, the same cortical and hippocampal regions active during memory retrieval are active during imagination (Schacter et al., 2008; Svoboda et al., 2006). It has therefore been theorized that previously formed memories – encoded by the frontal cortices and hippocampus – provide the building blocks that are used to imagine upcoming events (Buckner, 2010).

The hippocampus has long been associated with episodic memory (O’Keefe and Nadel, 1978; Scoville and Milner, 1957; Vargha-Khadem et al., 1997). In contrast, the prefrontal cortex has typically been linked to decision-making (Rushworth et al., 2011) and executive control (Posner et al., 2007). While the two structures were at one time thought to have mutually exclusive roles in cognition, this view was challenged by human imaging reports; neuroimaging studies demonstrate the co-activation of the medial prefrontal cortex (mPFC) and the hippocampus during a range of memory processes (Fletcher and Henson, 2001; Miyashita, 2004; Ranganath et al., 2004). Patients with damage to the mPFC show similar impairments with autobiographical memories (Bertossi et al., 2016; Fletcher and Henson, 2001) and imagination of future scenarios (Bertossi et al., 2017). Animal models also support the important contribution of the frontal cortex in a range of memory functions including with contextual fear memory (Frankland et al., 2004; Rozeske et al., 2018), odor reward association (Tronel and Sara, 2003), and spatial memory (Maviel et al., 2004). Together these studies support the view that both the hippocampus and the mPFC have integral roles in learning and memory.

The following review will discuss the roles of the hippocampus and the prefrontal cortex in memory functions and planning the future. We first provide a brief overview of the functional contributions of each region and their anatomical connectivity. We then discuss a key role for oscillatory synchrony that links neural representations of past, present, and future events. It should be noted, of course, that while human research can examine our capacity to imagine the future, research on the true sense of ‘imagination’ in animal models is not possible. We can however examine the neural representations of memory-based *planning*, which is closely linked to imagining the future and will be discussed here. Finally, we highlight a key circuit between the prefrontal cortex, nucleus reuniens (NRe), and hippocampus that may function to temporally segregate representations of alternate upcoming choices, which could reflect the planning of future trajectories.

## Prefrontal cortex-hippocampal pathways

**Figure 1.**
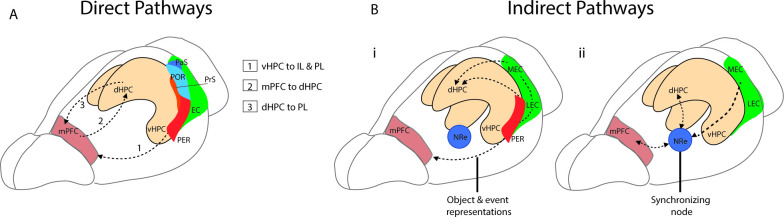
Direct and indirect pathways connecting the medial prefrontal cortex (mPFC) and the hippocampus. (**A**) Monosynaptic pathways between the both dorsal hippocampus (dHPC) and ventral hippocampus (vHPC) and prelimbic (PL) and infralimbic subregions of the mPFC with an anatomical overview of parahippocampal areas including presubiculum (PrS), parasubiculum (PaS), perirhinal (PER), and the postrhinal cortex (POR). (Bi) Bidirectional indirect pathways between the mPFC hippocampus passing through the PER and medial and lateral entorhinal cortices (MEC, LEC). (Bii) Bidirectional indirect mPFC-hippocampus pathway connecting through the nucleus reuniens (NRe).

The mPFC and hippocampus interact through several direct and indirect pathways. The first direct monosynaptic pathway to be described is a projection from the ventral and intermediate portion of CA1 and proximal subiculum. This projection innervates all layers of the mPFC and extends to the orbital portion of the PFC with the densest projections concentrated in the infralimbic (IL) and prelimbic (PL) regions (Cenquizca and Swanson, 2007; Hoover and Vertes, 2007; Jay et al., 1989; Figure 1A). The ventral portions of the hippocampus have been associated with a range of functions including fear (Kjelstrup et al., 2002), stress (Chang and Gean, 2019), anxiety (Jimenez et al., 2018), and social memory (Phillips et al., 2019). The ventral hippocampus (vHPC), like the dorsal portion, codes for spatial location, though place fields formed by ventral hippocampal neurons are considerably larger (Jung et al., 1994; Kjelstrup et al., 2008). Given its related functions and larger spatial scale, it is theorized that the vHPC transmits broad spatial and contextual information to the prefrontal cortices (Eichenbaum, 2017; Komorowski et al., 2013). Since this initial pathway was described, additional connections have been identified from the dorsal hippocampus (dHPC) which provides direct projections to the PL region of the mPFC, with activation of this pathway shown to mediate context fear memory retrieval (Ye et al., 2017). The mPFC in return sends direct monosynaptic projections to the hippocampus from the dorsal anterior cingulate portion of the mPFC to CA1 and CA3 subregions of the dHPC (Rajasethupathy et al., 2015), and from the PL to inhibitory neurons in dorsal CA1 (Malik et al., 2021, preprint) (Figure 1A).

Two of the main indirect pathways between the mPFC and the hippocampus pass either through the perirhinal and entorhinal cortices or through the NRe. One connection between the PL and IL and the hippocampus is a bidirectional pathway that passes through the perirhinal and entorhinal cortices to CA1 and CA3 of the hippocampus (Burwell et al., 1995; Lavenex et al., 2002; Vertes, 2004). The mPFC projects to both the superficial layers of the perirhinal cortex (PRC) as well as to the deep layers of both the lateral entorhinal cortex (LEC) and medial entorhinal cortex (MEC) (Agster and Burwell, 2009; Apergis-Schoute et al., 2006; Burwell and Amaral, 1998). This pathway to LEC and PRC is implicated in supporting the representations of objects and events (Deshmukh and Knierim, 2011; Eichenbaum, 2017; Kinnavane et al., 2016; Figure 1Bi). A second indirect pathway between the mPFC and the hippocampus passes through the thalamic subregion − the NRe (Vertes et al., 2007). The NRe sits along the midline of the thalamus and provides a bridge point between the prefrontal cortex and the hippocampal formation (McKenna and Vertes, 2004; Prasad and Chudasama, 2013; Vertes, 2006). Both the PL and IL provide dense projections to the NRe (McKenna and Vertes, 2004; Vertes, 2002; Vertes, 2004), and in turn, the NRe innervates many regions within the hippocampal formation including the entorhinal cortex (Wouterlood et al., 1990), presubiculum (McKenna and Vertes, 2004), parasubiculum (Wouterlood et al., 1990), and across the distal apical lamina of CA1 of the hippocampus (Bertram and Zhang, 1999). From the NRe, distinct populations of neurons project to the entorhinal cortex, CA1, and the subiculum, with entorhinal projections originating in the dorsolateral portion of the NRe, while more medial portions of the NRe project to CA1 (Dolleman-Van Der Weel and Witter, 1996). The NRe forms primarily excitatory synaptic connections to CA1 (Bertram and Zhang, 1999), and appears to innervate both pyramidal cells and putative interneurons (Dolleman-Van der Weel et al., 1997). Importantly, this pathway is bidirectional and functions as a link between the hippocampal formation and the mPFC. The NRe receives inputs from the subiculum and projects heavily to the PFC (Vertes, 2006). This link can be bypassed however as CA1, the subiculum and the EC also project directly to the PFC (Bertram and Zhang, 1999; Swanson and Kohler, 1986; Figure 1Bii).

The NRe neuron population is functionally heterogeneous, with hippocampal theta oscillations differentially modulating separate populations of neurons. Across the midline of the thalamus there are a high proportion of cells expressing both calretinin and calbindin, proteins known to regulate calcium homeostasis (Lara-Vasquez et al., 2016). Calretinin expressing cells do not alter their activity during bouts of theta but are inhibited by sharp-wave ripples (SWR). In contrast, cells lacking calretinin discharge at higher rates during hippocampal theta but are inactive during SWR activity (Lara-Vasquez et al., 2016).

Inactivation or lesions to the NRe result in memory deficits that resemble lesions to the mPFC and hippocampus, including with goal-directed spatial memory (Hallock et al., 2016; Hembrook et al., 2012; Layfield et al., 2015) and contextual fear memory (Ramanathan et al., 2018). In relation to hippocampal spatial coding, lesions to the NRe and the rhomboid nucleus of the thalamus do not impact hippocampal place cell characteristics in a familiar environment (Cholvin et al., 2018). However in novel environments, lesions induced place cell instability and high firing variability (Cholvin et al., 2018). The role of the NRe is therefore theorized to support long-term spatial stability and to synchronize the mPFC and hippocampus to facilitate memory processing (Cholvin et al., 2018; Ketz et al., 2015).

## Theta oscillations and the medial septum

One of the most prominent features of the hippocampus is the presence of strong oscillatory dynamics. The three main rhythms recorded in the hippocampus are theta (4–12 Hz) (Winson, 1974), SWR (~110–250 Hz ripples on ~125 ms sharp waves) (Buzsaki, 1986), and gamma (25–150 Hz) (Colgin et al., 2009). While each of the three main types of rhythms is functional distinct, the current review is focused on theta rhythms. Theta rhythms are essential to hippocampal-dependent functions including spatial navigation and memory (Winson, 1978). Disruptions to hippocampal theta results in significant deficits in spatial memory, which are often as severe as lesions to the hippocampus itself (O’Keefe and Nadel, 1978; Winson, 1978).

The medial septum (MS) is the central pacemaker for generating theta rhythms in the hippocampus and the entorhinal cortex (Colgin, 2013; Mizumori et al., 1990; Vertes and Kocsis, 1997). The MS consists of three separate populations: GABAergic, glutamatergic, and cholinergic neurons (Brashear et al., 1988; Kimura et al., 1980; Panula et al., 1980; Kohler et al., 1984; Shute and Lewis, 1967; Sotty et al., 2003). Each population of the MS has both distinct firing patterns and projection targets. Cholinergic neurons make up the largest portion of septal neurons with approximately 47% of the total MS population and are characterized by their slow-firing patterns (~5 Hz) (Colom et al., 2005; Griffith and Matthews, 1986; Markram and Segal, 1990). The majority of MS cholinergic neurons projections to the hippocampus innervate pyramidal cells and interneurons as well as some granule cells in the dentate gyrus (DG) ([Bibr bib30][Bibr bib51]; Widmer et al., 2006). In the entorhinal cortex, septal cholinergic neurons project more to the MEC than the LEC, 5% vs. 3.37% respectively, with most of the projections terminating in layers I and II (Desikan et al., 2018). Septal GABAergic neurons comprise approximately 28% of the total MS population (Colom et al., 2005). This population is characterized by its fast-firing and burst firing patterns as well as the presence of Ih hyperpolarization-activated current. Hyperpolarization-activated currents are inward currents that are activated by the hyperpolarizing of cells and are found in rhythmically active neurons (Hangya et al., 2009; Varga et al., 2008). MS GABAergic neurons form local projections within the septum with cholinergic and other GABAergic neurons (Henderson et al., 2001) as well as long-range projections to both the hippocampus and the medial entorhinal cortex (Gulyás et al., 1991; Henderson et al., 2001; Gonzalez-Sulser et al., 2014). The population can be subdivided into at least two separate populations, those cells positive for the parvalbumin (PV) marker and cells positive for calbindin marker (Kiss et al., 1997). MS GABAergic neurons project to CA1, CA3, and the DG subregions of the hippocampus and primarily innervate other GABAergic interneurons (Freund and Antal, 1988). Similar to the projection pattern in the hippocampus, the MS GABAergic population primarily projects to interneurons across all layers of the MEC including both fast-spiking and low threshold spiking interneurons (Gonzalez-Sulser et al., 2014).

Septal glutamatergic neurons make up approximately 25% of the MS population (Colom et al., 2005). MS glutamatergic neurons provide excitatory inputs to cholinergic, GABAergic, and other glutamatergic neurons within the MS (Manseau et al., 2005; Robinson et al., 2016), with the majority of projections to fast-spiking putative GABAergic neurons (Robinson et al., 2016). Next, these neurons constitute between 4% and 23% of the septo-hippocampal projections (Colom et al., 2005; Henderson et al., 2010) with projections to both interneurons and pyramidal cells in CA1 and CA3 (Fuhrmann et al., 2015; Robinson et al., 2016). MS glutamatergic neurons also send direct projections to the MEC and appear to preferentially project to pyramidal cells in the MEC (Gonzalez-Sulser et al., 2014; Justus et al., 2017).

Lesions or inactivation of the entire MS abolishes theta oscillations in the hippocampus and entorhinal cortex (Green and Arduini, 1954; Jeffery et al., 1995; Mitchell et al., 1982), and recordings from septal neurons display rhythmic burst firing patterns that are synchronized to the ongoing hippocampal theta oscillation (Stumpf et al., 1962). Each separate MS population contributes differently to the rhythm generation. MS GABAergic neurons are hypothesized to drive theta oscillations by providing rhythmic inputs to local interneurons in the hippocampus (Freund and Antal, 1988). These interneurons then rhythmically disinhibit pyramidal cells in the hippocampus therefore promoting widespread and synchronized theta oscillation. Rhythmic optogenetic stimulation of septal GABAergic neurons drives hippocampal oscillations with a high level of entrainment within the theta range (Zutshi et al., 2018) and optogenetic silencing of GABAergic neurons significantly reduced theta power both during REM sleep (Boyce et al., 2016). Septal cholinergic neurons have been reported to control the amplitude of ongoing theta rhythms (Lee et al., 1994). Despite these findings, recent chemogenetic and optogenetic experiments suggest that modulation of septal cholinergic neurons may have more moderate effects on hippocampal theta rhythms than previously reported. Chemogenetic activation of septal cholinergic neurons moderately reduces theta rhythm frequency within the MEC (Carpenter et al., 2017), and optogenetic stimulation of septal cholinergic neurons at theta frequencies promotes theta rhythms during anesthesia, however has little effect in the awake and behaving mouse (Vandecasteele et al., 2014). Septal glutamatergic cells may contribute to theta rhythm generation, as rhythmic optogenetic activation of septal glutamatergic neurons within the theta range strongly drives theta rhythms in the hippocampus (Fuhrmann et al., 2015; Robinson et al., 2016). Activation of glutamatergic neurons also results in locomotor initiation and have therefore been suggested to mediate transition states associated with locomotion (Fuhrmann et al., 2015). The direct contribution of septal glutamatergic neurons to the generation of theta oscillations or movement transitions remains to be determined, as it has yet to be reported if silencing this population has any effect on theta oscillations or movement.

## Oscillatory synchrony and the NRe

Across the brain, a proposed function for neural oscillations is to facilitate long-range communication between brain areas. In both rodent and human studies, theta coherence between the mPFC and the hippocampus has been related to both working memory (Anderson et al., 2010; Benchenane et al., 2010; Hyman et al., 2005) and goal-directed spatial navigation (Ito et al., 2015). During goal-directed spatial navigation tasks, many mPFC cells become phase-locked to hippocampal theta rhythms (Hyman et al., 2005; Siapas et al., 2005). In simultaneous recordings of units in the mPFC and field activity from CA1, a high level of coherence between the two regions is present at the choice point in a Y-maze task (Benchenane et al., 2010). This choice point-related coherence increases following acquisition of the task (Benchenane et al., 2010). A decrease in the phase-locking between the two regions is also predictive of errors during cognitive testing (Hyman et al., 2010).

The NRe is key in supporting synchrony between the mPFC and the hippocampus across frequency bands. Inactivation of the NRe disrupts gamma synchrony between the hippocampus and mPFC during urethane anesthesia (Ferraris et al., 2018). In addition, during the choice point of a delayed alternation T-maze task, muscimol infusion into the NRe results in a significant decrease in both mPFC spike phase-locking to theta and a decrease in oscillatory coherence between the two regions (Hallock et al., 2016). The importance of the NRe is highlighted during a spatial alternation T-maze task, where recordings from the mPFC and NRe show that the firing rates of neurons predict future turns at the choice point (Ito et al., 2015). This predictive and trajectory-dependent activity during the stem portion of the T-maze is abolished during optogenetic silencing or lesions to the NRe (Ito et al., 2015). These studies support the theory that the NRe is critically involved in synchronizing the mPFC and the hippocampus during periods when the animal could be contemplating an upcoming decision (Ito et al., 2015; Eichenbaum, 2017). Further support for the role of the NRe in hippocampal-related memory processing comes from a study that used optogenetic activation of the NRe, which resulted in an enhancement for memory performance during a context conditioning task (Xu and Sudhof, 2013). It should be noted with many direct and indirect pathways, theta coherence may be in part regulated through other connections, for example, the direct vHPC to mPFC connections also contribute to theta synchrony between the two regions (O’Neill et al., 2013). Together these studies demonstrate the importance of theta synchrony in supporting memory performance and highlights the role of the NRe as a bridge point between the mPFC and the hippocampus to facilitate bidirectional communication.

## Hippocampal representations are organized within theta cycles

**Figure 2.**
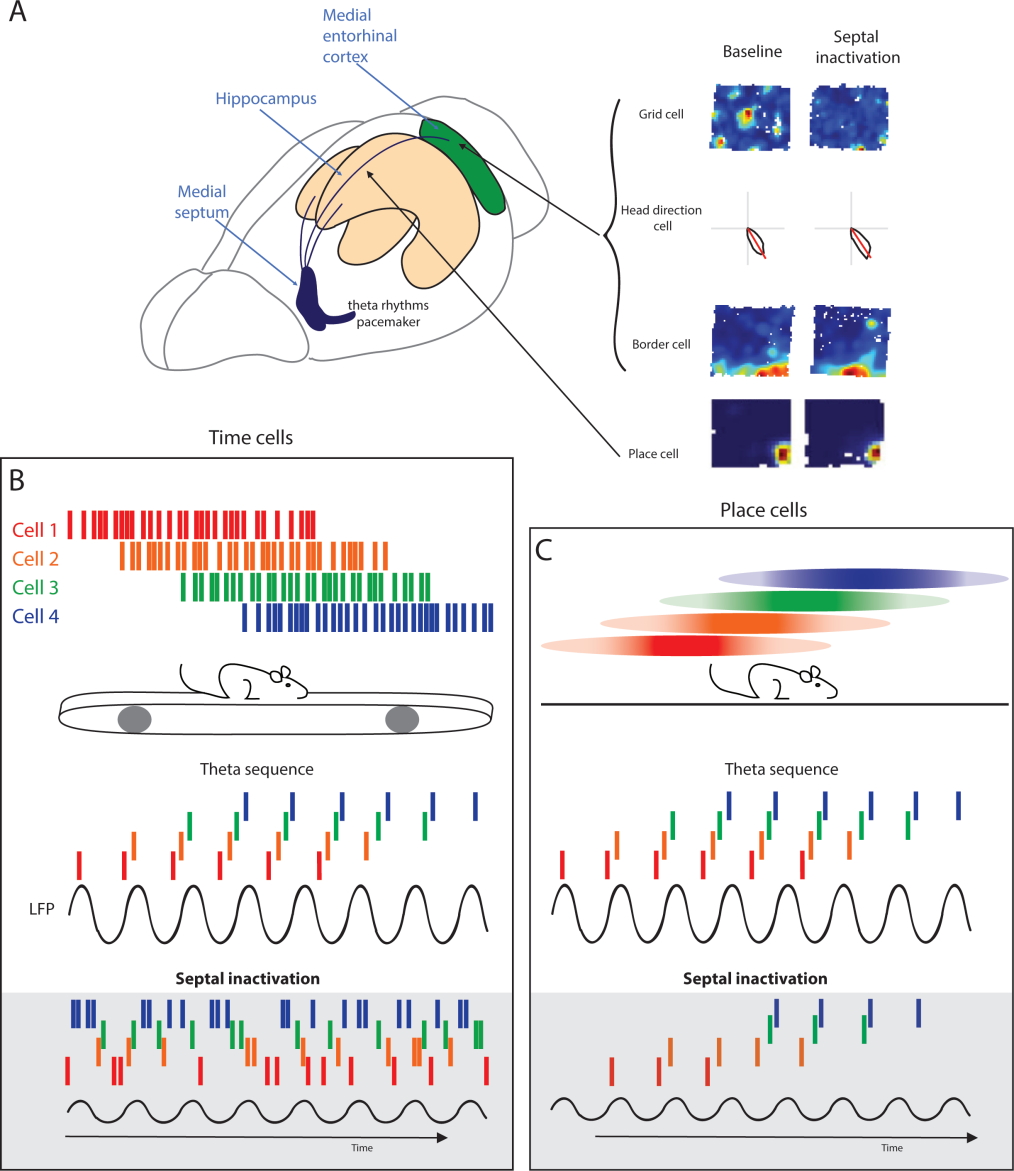
Alternating theta cycles provide a framework for imagining or deliberating between future options. (**A**) Diagram of the hippocampal formation with examples different cell types recorded across the hippocampus and the medial entorhinal cortex (MEC). (**B**) Hippocampal time cell sequences in relation to hippocampal theta (above) and during septal inactivation (below). (**C**) Hippocampal place cell sequences in relation to hippocampal theta (above) and during septal inactivation (below).

Within the hippocampus, theta rhythms provide a temporal framework to organize spatial information. The hippocampus creates a distinctive spatial representation of its environments, in the form of populations of ‘place cells’, that form an internal map of the environment and are hypothesized to enable spatial memory (Burgess et al., 2001; Buzsaki and Moser, 2013). In relation to individual cycles of the theta rhythm, the initial firing of a place cell occurs at a late phase of the theta cycle − at the peak of the oscillation − but as the animal traverses through the place field the cell fires at progressively earlier phases within each subsequent theta cycle, known as phase precession (O’Keefe and Recce, 1993; Skaggs et al., 1996). At the population level, a sequence of place cells within each theta cycle will spike in the same order that place fields were visited, in a time-compressed format (Lee and Wilson, 2002; Skaggs et al., 1996; Figure 2). One important consequence of this temporal organization is that place fields centered behind the animal will fire first in the sequence, at the earliest theta phase. Likewise, cells with a field centered in front of the animal will fire at later phases. The temporal organization of theta sequences therefore represent the immediate past, present, and future of the animal’s location within a theta cycle (Figure 2C; Skaggs et al., 1996).

Representations of past and potential future experiences are not only present during active navigation, but these sequences are also observed with replay during SWR. SWR occur during ‘offline’ states of the animal, including during non-REM sleep, immobility, as well as eating and drinking (Buzsaki, 1986). Although this phenomenon may play an essential role in memory formation and future decision-making (Buzsáki, 2015; Foster, 2017; Joo and Frank, 2018; Shin et al., 2019), this review will focus on hippocampal representations during active navigation.

Phase precession and theta sequences are related but have distinct phenomena. Theta sequences emerge following experience, while phase precession can be recorded during the first time traversing through a novel environment (Feng et al., 2015). It was therefore theorized that phase precession is a building block of theta sequences, with theta sequences being formed by synchronizing multiple neurons into coherent ensembles through experience-dependent plasticity (Feng et al., 2015). However, recent reports show that the two phenomena are independent from one another. Following modulation of cholinergic tone, phase precession is disrupted while hippocampal ensembles remain intact (Venditto et al., 2019). Furthermore, the reduction of hippocampal theta oscillations does not affect place cell phase precession (Schlesiger et al., 2015), but will eliminate theta sequences in both time cells and place cells (Figure 2B and C; Wang et al., 2015).

Phase precession has recently been identified in human subjects during goal-directed navigation tasks. In recordings performed in a virtual environment, theta oscillations in CA1 are present at a broader range compared to rodent theta (2–10 Hz) (Qasim et al., 2021). Recordings from across the hippocampus and the entorhinal cortex demonstrate spatially tuned neurons spike at progressively earlier phases of the theta oscillation while an individual navigates through the cells’ firing field (Qasim et al., 2021). In addition, there is evidence of phase precession in non-spatially modulated cells during specific goal states. This phase precession is present in the hippocampus as well as in the anterior cingulate and orbitofrontal cortex. This goal-specific phase precession suggests that phase precession may be functionally relevant for neural representations of both spatial and non-spatial information across both the medial temporal and prefrontal cortex (Qasim et al., 2021).

Many questions remain regarding theta sequences including how are these spatial and temporal patterns formed and if these temporally precise patterns are necessary for memory formation. For insights into these questions, we turn to one of the main input pathways into the hippocampus, the MEC. The MEC contains a variety of spatially modulated neurons that include grid cells (Hafting et al., 2005), border cells, (Solstad et al., 2008), and head direction (HD) cells (Sargolini et al., 2006). Since the identification of grid cells, it was widely theorized that inputs from the MEC and grid cells support the formation of place cells in the hippocampus (Buzsaki and Moser, 2013; Cheng and Frank, 2011; de Almeida et al., 2012; McNaughton et al., 2006; Moser and Moser, 2013; Rolls et al., 2006; Savelli and Knierim, 2010; Si and Treves, 2009). Despite these theories, however, it was shown that inactivation of the MS and the attenuation of theta rhythms completely abolishes grid cell spatial firing (Brandon et al., 2011; Koenig et al., 2011) while hippocampal place cells remain unaffected in both familiar and novel environments (Brandon et al., 2014; Koenig et al., 2011). Thus, the retrieval of previously stored spatial maps, as well as the generation of new spatial maps, is not dependent on inputs from the MS nor from MEC grid cells. This relationship between grid cells and place cells is further supported by early recordings in rat pups, which show that place cell spatial firing develops days earlier (P15-17) than grid cells (P20-22) (Langston et al., 2010; Wills et al., 2010). Even though grid cells are not essential to support place cell spatial firing, other inputs from the MEC are necessary as shown by lesions to the entire EC disrupts both the temporal precision and the spatial stability of place cells (Miller and Best, 1980; Schlesiger et al., 2018; Van Cauter et al., 2008).

Given that the disruption of the MS and the reduction in theta oscillations results in strong deficits in spatial memory performance, the role of septal inputs to the hippocampus has been theorized to temporally coordinate cell assemblies to enable memory formation (Buzsaki and Moser, 2013) and may also provide a framework for the representation of future possible trajectories. Recent work has provided insight into how the septum supports this temporal coordination. The complete MS inactivation and theta reduction results in the attenuation of theta sequences in both time cells and place cells while phase precession is maintained (Figure 2B and C; Wang et al., 2015). With a more moderate alternation to MS activity, by cooling the septum, the temporal organization of hippocampal place cells exhibited less of a disruption. Cooling of the MS reduced both theta frequency and power, leaving theta oscillations intact but slowed during a T-maze task (Petersen and Buzsaki, 2020). Slowing theta oscillations did not affect the distance between place cell peaks or the phase lag time but the presence of an increased time lag suggested that the same number of place cell assemblies were compressed into a theta cycle, however, the assemblies were proportionally altered within the lengthened theta cycle during MS cooling (Petersen and Buzsaki, 2020). Different populations of the MS have distinctive contributions to the temporal coding of information in the hippocampus. As discussed earlier, the systemic modulation of cholinergic neurons results in the disruption of phase precession, however, theta sequences were maintained (Venditto et al., 2019). In addition, optogenetic activation of MS PV neurons to drive hippocampal rhythms outside the endogenous range (10–12 Hz) does not alter place cell spatial firing nor cell phase precession (Zutshi et al., 2018). Together these studies indicate that the MS is not required for temporal coordination of individual cells relative to theta oscillations (phase precession); however, the timing between hippocampal place cells is altered, suggesting that MS inputs are essential for the generation of theta sequences.

Recordings in developing rat pups further supports this relationship between the MS, theta oscillations, and sequences. During development in rat pups, the spatial coding of hippocampal place cells appears as early as P15 (Farooq and Dragoi, 2019; Langston et al., 2010; Wills et al., 2010) and phase precession in these cells appears shortly thereafter, as early as P17 (Wills et al., 2010). However, temporal organization between cells, or theta sequences that represent previous, present, and future locations, do not develop until considerably later, at P23-24. Thus at the earliest stages of the spatial coding network, the hippocampus represents the animal’s current location only, whereas representations that extend into the immediate past and future are not present until P23-24 (Farooq and Dragoi, 2019). Of course, many important features of the network are rapidly maturing between P15 and P24. Here, we highlight the development of the MS theta generation circuit as an important candidate to support theta sequence development. In the MS, neurons begin to express PV neurons at P16, however, very few neurons are present at this stage (Guo et al., 2014). Between P16 and P21 there is a rapid development of this cell type, with numbers of PV positive immunoreactive cells nearly tripling during this time and then reaching close to fully mature levels by P28 (Guo et al., 2014). In addition, peak theta frequency increases with age along with the proportion of CA1 and MEC cells that are theta-modulated (Farooq and Dragoi, 2019; Wills et al., 2010). The development of theta sequences also corresponds with the maturation of the MEC and the emergence of grid cell spatial firing (P20-22) (Langston et al., 2010; Wills et al., 2010), which may enable the appearance of theta sequences in CA1 (Farooq and Dragoi, 2019). If we use PV development and hippocampal theta as indicators of MS maturation, the MS and theta oscillations are critical components for the emergence of MEC grid cells and hippocampal theta sequences. The emergence of MEC grid cells may support hippocampal theta sequences through path integration. For hippocampal place cells to code for both past and future trajectories while the animal navigates through an environment, the hippocampus needs to integrate multiple sensory inputs and self-generated cues by the animal’s movement for both retrospective and prospective coding. Grid cells may play a pivotal role in theta sequence generation by providing idiothetic cues for path integration. Path integration is a form of navigation that relies on the continuous integration of self-motion information about distance traveled and running speed. The regular hexagonal firing pattern of grid cells may support prospective coding through the integration of self-motion-based information chunked into a metric of space.

The maturation of hippocampal theta, the development of grid cells, and the emergence of theta sequences all coincide with the successful acquisition of hippocampal-dependent learning and memory tasks. In the Morris water maze, evidence for place learning can occur as early as P19-20 (Brown and Whishaw, 2000), but quadrant preference during the probe trial develops at a similar age as the emergence of theta sequences (P20-21) (Akers and Hamilton, 2007). Similarly, rat pup performance exceeds chance levels in an alternation T-maze at P21-25 (Bronstein and Spear, 1972), corresponding to when theta sequences emerge. Together, these developmental studies and others strongly suggest that the maturation of the MS to drive theta rhythms and development of grid cells may be essential for temporal coding of theta sequences, enabling the representation of past, present, and future trajectories.

In addition to developmental studies, there are several lines of evidence that support the theory that the temporal coding and binding of successive locations into theta sequences is essential for both memory formation and representing future events. Theta sequences are more frequently represented between landmarks in an environment separating the environment into segments (Gupta et al., 2012). Therefore, the hippocampus may encode spatial information into segments of experience, suggesting that the brain organizes vast amounts of information in an efficient and useful way (Gupta et al., 2012). Theta sequences are absent when navigating through an environment for the first time (Feng et al., 2015). Following experience in an environment, theta sequences emerge, predicting the immediate future of the animal path (Feng et al., 2015; Pfeiffer and Foster, 2013). These studies support the notion that theta sequences play roles in both consolidating past experiences while planning for the future.

## Sequence alternation and the representation of future scenarios

To represent past and future trajectories, hippocampal place cells display both prospective and retrospective coding. Initial reporting showed that during a modified T-maze task hippocampal place cells display differential firing during the stem portion of the maze, depending on the animals upcoming trajectory (Wood et al., 2000). Later studies reveal that during goal-directed spatial navigation, cell assembles exhibit both prospective coding of the upcoming path and retrospective coding of where the animal has been (Ferbinteanu and Shapiro, 2003; Wang et al., 2020). Recently, it has been shown that within a single theta cycle, hippocampal place cell sequences alternate between forward and reverse ordered sequences (Wang et al., 2020). Forward sequences have a preferred phase around 250–420°, while the reverse phase occurs between 80° and 230° of the theta cycle (where 0° is the peak of theta). Interestingly, the reverse ordered sequences appear very similar to reverse replay which can be recorded during SWR (Wang et al., 2020), and thus may facilitate the expression of reverse replay and memory consolidation.

**Figure 3.**
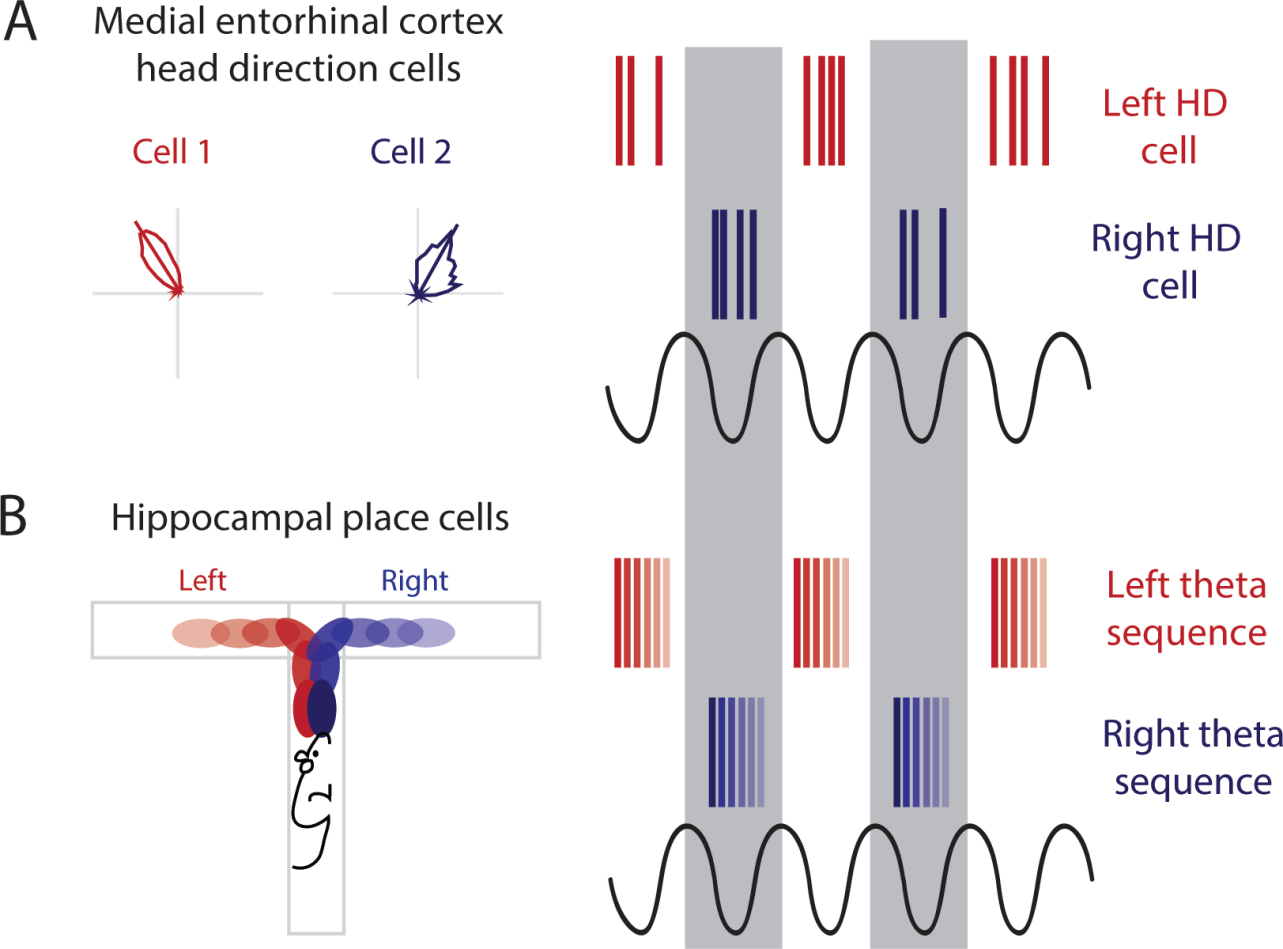
Alternating theta cycles provide a framework for alternate representations of future options. (**A**) Head direction cell assemblies on alternating theta cycles recorded in the medial entorhinal cortex (MEC). (**B**) From inputs received from the MEC may support theta sequences of upcoming potential paths on alternating theta cycles.

Alternation between sequences is also present when two possible paths lie ahead. During a T-maze, when two trajectories are possible leading up to the choice point of a maze, hippocampal theta sequences map out the future paths that correspond to potential future actions, indicating the animal could be deliberating between two choices (Johnson and Redish, 2007; Kay et al., 2020). This mapping of potential future paths is organized into alternating theta cycles (Kay et al., 2020). In the MEC, HD cells segregate onto alternating theta cycles (Brandon et al., 2013; Jeffery et al., 1995). This phenomenon is lost when theta rhythms are disrupted through MS inactivation, pointing toward theta oscillations as a means to segregate discrete information in time by assigning this information to alternating cycles of the theta rhythm (Figure 3A; Brandon et al., 2013). Along these same lines, during a T-maze task, the hippocampus will represent both options (left or right) when the animal reaches the choice point; first sweeping forward along one potential path and then the other, possibly evaluating both options (Johnson and Redish, 2007). More recently, it has been shown that prior to this left-right decision at a choice point, CA1 cells that encode the left choice will be selectively active on one theta cycle followed by cells that encode the right choice will then be active on the next theta cycle (alternating every ~125 ms) (Figure 3B; Kay et al., 2020). This alternation between left and right begins as early as 25 cm prior to the choice point and will continue until the animal makes its turn (Kay et al., 2020). When the hippocampal representation alternates between two possible future trajectories or choices, this organization may be driven by theta cycles and inputs received from theta alternating cells in the MEC (Figure 3). Further evidence for theta skipping can be found across several regions that provide input to the MEC including (1) the MS where theta skipping neurons are also visible in directionally tuned cells that are only theta rhythmic when the animal runs in a specific direction (King et al., 1998), and (2) the pre- and parasubiculum where theta skipping can be seen in HD and directionally modulated ‘border cells’ (Boccara et al., 2010).

Representations of past, present, and future trajectories have most recently been recorded in the PFC (Tang et al., 2021). PFC cell ensembles are organized into sequences within a single theta cycle that occur concurrently with hippocampal sequences. During the W-maze spatial alteration task, approximately half of the sequences recorded in the PFC and CA1 exhibit theta cycle skipping. Interestingly, while the animal navigates toward the choice point, CA1, but not PFC, theta sequences alternate between possible upcoming trajectories. Instead, PFC sequences preferentially encode the animals’ upcoming choice (Tang et al., 2021). It appears that the hippocampus equally represents both potential paths, while the PFC will represent the future choice earlier, with CA1-PFC theta sequence coherence between the two regions biased toward the upcoming choice. Therefore, it is suggested that the hippocampus interacts with the prefrontal sequences to guide the selection of an upcoming choice.

**Figure 4.**
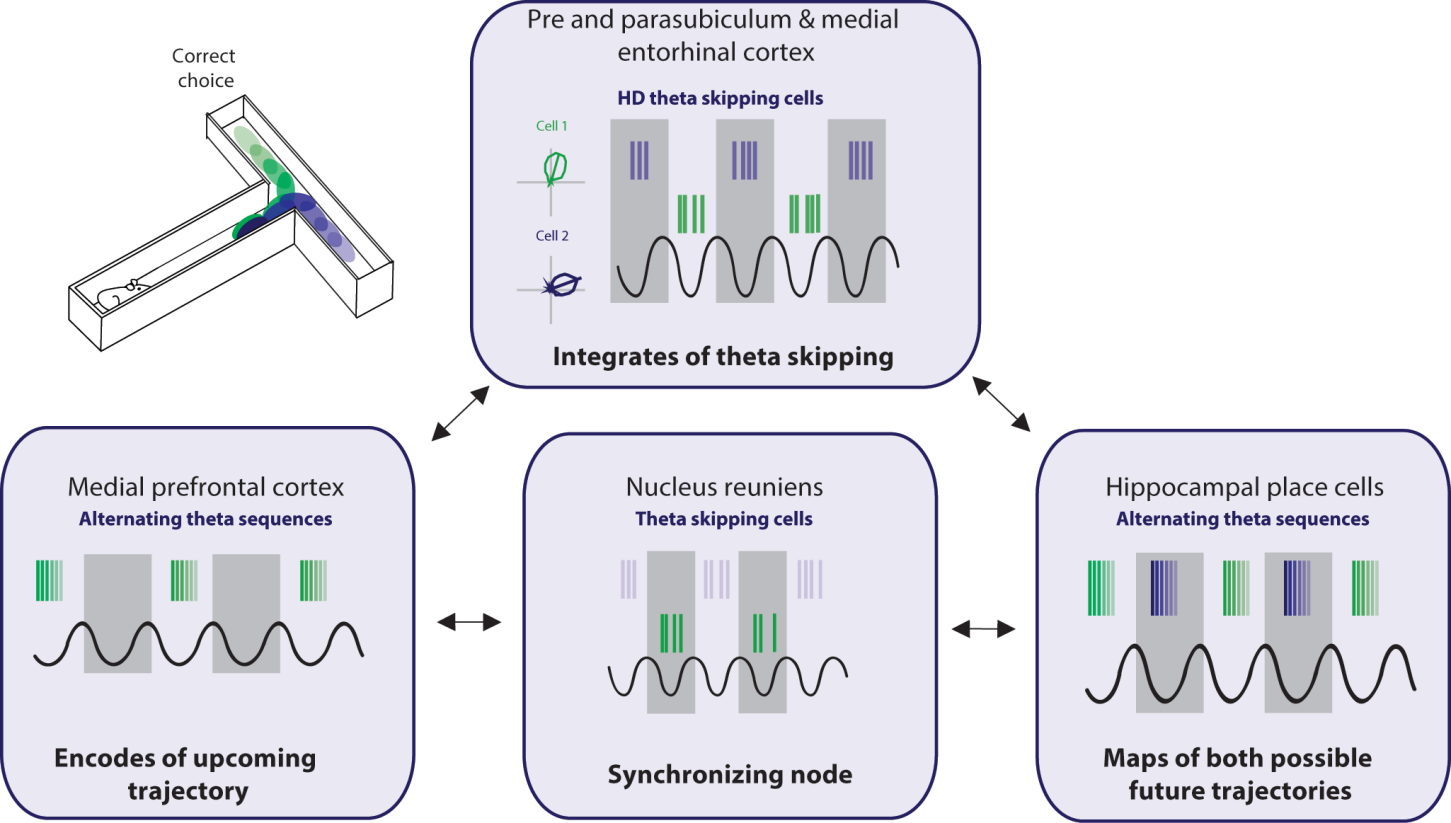
Proposed circuit for representation of future scenarios. The medial prefrontal cortex (mPFC) and nucleus reuniens (NRe) provide unique input to the pre- and parasubiculum, and medial entorhinal cortex on alternating theta cycles. The pre- and parasubiculum and the medial entorhinal cortex (MEC) represent head directions to the animal’s right and left on alternating theta cycles. In turn, these inputs drive the expression of alternating theta sequences that map out future scenarios in both the hippocampus and mPFC.

As discussed above, the interaction between the mPFC and the hippocampus through the NRe during a spatial memory task is critical, particularly leading up to the decision point. Since this is the portion of the task when future sequences are being actively represented, the interaction between the PFC and hippocampus is of particular importance. During exploration in an open field environment, the NRe contains one population of cells that codes for HD and a separate population that are ‘purely’ theta cycle skipping (Jankowski et al., 2014). While these cells are in separate populations in the NRe, this information may be integrated further down the line at the level of the pre- and parasubiculum and the MEC (Figure 4). During spatial navigation, the NRe supports the animals’ future trajectory firing in CA1, however, this trajectory-dependent firing is lost with NRe lesioning or optogenetic shutdown (Ito et al., 2015). We propose that this loss is due to the inactivation of theta alternating inputs to the pre- and parasubiculum and the MEC. In this model, the central role of the MS is to drive theta rhythms and provide a time course to segment incoming sensory information in the parahippocampal regions, and the role of the NRe is to provide input that separates this information into adjacent theta cycles. Together, theta cycle spiking would be first integrated at the level of the pre- and parasubiculum and the MEC. The pre- and parasubiculum and the MEC represent HD to the animal’s right and left on alternating theta cycles. In turn, these inputs drive the expression of alternating theta sequences that map out future scenarios in both the hippocampus and mPFC to guide the animals’ upcoming choices and behavior. It should be noted that to date cycle skipping has not to be linked to any type of behavioral correlate, we theorize that theta skipping in parahippocampal areas plays a key role in supporting the segmentation of different spatial representations to enable the representation of multiple possible of future trajectories.

While the mechanisms outlined in the current review are focused on spatial representations, this circuit can be extended to non-spatial tasks as well. Spatial representation is one example of a more general mechanism for the encoding of salient variables by the hippocampus (Buzsaki and Moser, 2013; Constantinescu et al., 2016; Schiller et al., 2015; Tavares et al., 2015). Although the data presented here has been primarily focused on spatial representations, firing of hippocampal cells can be modulated by a range of non-spatial variables including time (Wang et al., 2015), sound (Aronov et al., 2017; Sakurai, 2002), odors (Eichenbaum et al., 1987), faces, and objects (Fried et al., 1997). Given that hippocampal theta sequences have been observed in relation to time cells, and that these cells can be modulated by MS modulation, we believe this phenomenon can extend to any variable coded for by the hippocampus.

Looking more broadly, we predict that theta cycle skipping plays an important role in multiple functions including in deliberative decision-making behaviors. When an animal is navigating through an environment and comes to a decision point, the animal sometimes pauses and looks before executing the choice, seemingly deliberating between options, known as ‘vicarious trial and error’ (VTE) (Muenzinger, 1956; Tolman, 1948). During VTE, it has been suggested that the animal may be ‘thinking about the future’ (Redish, 2016). Further research could aim to unpack the relationship between alternating theta sequences and VTE behavior. This could be achieved through a detailed analysis of the animals’ running speed, head movement, and eye saccades with simultaneously recorded theta sequences. Recent reports have demonstrated the use of head-mounted camera to capture eye movements in freely moving rodents, allowing the possibility to examine rodent head and bilateral eye movement (Meyer et al., 2020). Using this technique during a goal-directed task, we predict that the precise analysis of the animals’ behavior, specifically of the animals’ head movement and saccades, would correspond to the theta skipping content in the prefrontal cortex and the hippocampus.

The mechanism discussed here refers to representations when only two possible outcomes are available, however, this alternation of cell assembles may also occur when multiple outcomes are possible. When there are two possible outcomes, for example, as an animal runs toward the choice point of a T-maze, each possible outcome remains ahead of the animal and can be represented on alternate theta cycles. These separate populations of neurons may interact competitively via lateral inhibition. Thus, alternating theta cycles provides a mechanism to prevent interference between attractor states while the animal deliberates. While the phenomenon of cycle skipping has been discussed here as deliberation between with two options performing in a T-maze, choices are often quite numerous. When there are multiple potential outcomes, for example, with a radial arm maze, an animal is presented with many available paths ahead. When this occurs, multiple attractor states may be present representing the potential paths. While the animal deliberates, theta sequence representation may occur intermittently and would likely be represented on separate theta cycles sporadically, perhaps not initially on alternating theta cycles. One possibility is that, as lateral inhibition of the competing attractors occurs, the hippocampus would eventually narrow down the potential path to two possible outcomes. At this point, we envision that the hippocampus would then represent the two possible attractor states on alternating cycles to drive the activation of cortex-wide representations relevant to the upcoming decision faced by the animal. Such a mechanism would allow the animals to explore previously encoded information to help consider the possible consequences of each decision.

### Concluding Remarks

Theta sequences may not only enable memory formation but also map out future potential paths. Representations of different outcomes are supported by segregation of content of these paths into alternating theta cycles in the hippocampus, which then allows for the final choice to be represented in the PFC. We propose that sequence representation on alternating theta cycles in both the PFC and the hippocampus is driven from inputs received from the parahippocampal regions including the presubiculum, parasubiculum, and the MEC. Furthermore, we suggest that to facilitate theta cycle skipping, as the animal weighs out two potential choices, requires the convergence of several regions that merge within the parahippocampal regions, and then the hippocampus and PFC. Specifically, inputs from the NRe, together with theta oscillations driven by the MS, are integrated at the level of the pre- and parasubiclulum and/or MEC. We propose that theta cycle skipping enables the mPFC and the hippocampus to segment unique possible outcomes, providing a framework for envisioning the future to guide behavior.

## References

[bib1] Agster KL, Burwell RD (2009). Cortical efferents of the perirhinal, postrhinal, and entorhinal cortices of the rat. Hippocampus.

[bib2] Akers KG, Hamilton DA (2007). Comparison of developmental trajectories for place and cued navigation in the Morris water task. Developmental Psychobiology.

[bib3] Anderson KL, Rajagovindan R, Ghacibeh GA, Meador KJ, Ding M (2010). Theta oscillations mediate interaction between prefrontal cortex and medial temporal lobe in human memory. Cerebral Cortex.

[bib4] Apergis-Schoute J, Pinto A, Pare D (2006). Ultrastructural organization of medial prefrontal inputs to the rhinal cortices. The European Journal of Neuroscience.

[bib5] Aronov D, Nevers R, Tank DW (2017). Mapping of a non-spatial dimension by the hippocampal-entorhinal circuit. Nature.

[bib6] Benchenane K, Peyrache A, Khamassi M, Tierney PL, Gioanni Y, Battaglia FP, Wiener SI (2010). Coherent theta oscillations and reorganization of spike timing in the hippocampal- prefrontal network upon learning. Neuron.

[bib7] Bertossi E, Tesini C, Cappelli A, Ciaramelli E (2016). Ventromedial prefrontal damage causes a pervasive impairment of episodic memory and future thinking. Neuropsychologia.

[bib8] Bertossi E, Candela V, De Luca F, Ciaramelli E (2017). Episodic future thinking following vmPFC damage: Impaired event construction, maintenance, or narration. Neuropsychology.

[bib9] Bertram EH, Zhang DX (1999). Thalamic excitation of hippocampal CA1 neurons: a comparison with the effects of CA3 stimulation. Neuroscience.

[bib10] Boccara CN, Sargolini F, Thoresen VH, Solstad T, Witter MP, Moser EI, Moser MB (2010). Grid cells in pre- and parasubiculum. Nature Neuroscience.

[bib11] Boyce R, Glasgow SD, Williams S, Adamantidis A (2016). Causal evidence for the role of REM sleep theta rhythm in contextual memory consolidation. Science.

[bib12] Brandon MP, Bogaard AR, Libby CP, Connerney MA, Gupta K, Hasselmo ME (2011). Reduction of theta rhythm dissociates grid cell spatial periodicity from directional tuning. Science.

[bib13] Brandon MP, Bogaard AR, Schultheiss NW, Hasselmo ME (2013). Segregation of cortical head direction cell assemblies on alternating theta cycles. Nature Neuroscience.

[bib14] Brandon MP, Koenig J, Leutgeb JK, Leutgeb S (2014). New and distinct hippocampal place codes are generated in a new environment during septal inactivation. Neuron.

[bib15] Brashear HR, Godec MS, Carlsen J (1988). The distribution of neuritic plaques and acetylcholinesterase staining in the amygdala in Alzheimer’s disease. Neurology.

[bib16] Bronstein PM, Spear NE (1972). Acquisition of a spatial discrimination by rats as a function of age. Journal of Comparative and Physiological Psychology.

[bib17] Brown RW, Whishaw IQ (2000). Similarities in the development of place and cue navigation by rats in a swimming pool. Developmental Psychobiology.

[bib18] Buckner RL (2010). The role of the hippocampus in prediction and imagination. Annual Review of Psychology.

[bib19] Burgess N, Becker S, King JA, O’Keefe J (2001). Memory for events and their spatial context: models and experiments. Philosophical Transactions of the Royal Society of London. Series B, Biological Sciences.

[bib20] Burwell RD, Witter MP, Amaral DG (1995). Perirhinal and postrhinal cortices of the rat: a review of the neuroanatomical literature and comparison with findings from the monkey brain. Hippocampus.

[bib21] Burwell RD, Amaral DG (1998). Cortical afferents of the perirhinal, postrhinal, and entorhinal cortices of the rat. The Journal of Comparative Neurology.

[bib22] Buzsaki G (1986). Hippocampal sharp waves: their origin and significance. Brain Research.

[bib23] Buzsaki G, Moser EI (2013). Memory, navigation and theta rhythm in the hippocampal-entorhinal system. Nature Neuroscience.

[bib24] Buzsáki G (2015). Hippocampal sharp wave-ripple: A cognitive biomarker for episodic memory and planning. Hippocampus.

[bib25] Carpenter F, Burgess N, Barry C (2017). Modulating medial septal cholinergic activity reduces medial entorhinal theta frequency without affecting speed or grid coding. Scientific Reports.

[bib26] Cenquizca LA, Swanson LW (2007). Spatial organization of direct hippocampal field CA1 axonal projections to the rest of the cerebral cortex. Brain Research Reviews.

[bib27] Chang CH, Gean PW (2019). The Ventral Hippocampus Controls Stress-Provoked Impulsive Aggression through the Ventromedial Hypothalamus in Post-Weaning Social Isolation Mice. Cell Reports.

[bib28] Cheng S, Frank LM (2011). The structure of networks that produce the transformation from grid cells to place cells. Neuroscience.

[bib29] Cholvin T, Hok V, Giorgi L, Chaillan FA, Poucet B (2018). Ventral Midline Thalamus Is Necessary for Hippocampal Place Field Stability and Cell Firing Modulation. The Journal of Neuroscience.

[bib30] Cole AE, Nicoll RA (1983). Acetylcholine mediates a slow synaptic potential in hippocampal pyramidal cells. Science.

[bib31] Colgin LL, Denninger T, Fyhn M, Hafting T, Bonnevie T, Jensen O, Moser MB, Moser EI (2009). Frequency of gamma oscillations routes flow of information in the hippocampus. Nature.

[bib32] Colgin LL (2013). Mechanisms and functions of theta rhythms. Annual Review of Neuroscience.

[bib33] Colom LV, Castaneda MT, Reyna T, Hernandez S, Garrido-Sanabria E (2005). Characterization of medial septal glutamatergic neurons and their projection to the hippocampus. Synapse.

[bib34] Constantinescu AO, O’Reilly JX, Behrens TEJ (2016). Organizing conceptual knowledge in humans with a gridlike code. Science.

[bib35] de Almeida L, Idiart M, Lisman JE (2012). The single place fields of CA3 cells: a two-stage transformation from grid cells. Hippocampus.

[bib36] Deshmukh SS, Knierim JJ (2011). Representation of non-spatial and spatial information in the lateral entorhinal cortex. Frontiers in Behavioral Neuroscience.

[bib37] Desikan S, Koser DE, Neitz A, Monyer H (2018). Proceedings of the National Academy of Sciences of the United States of America Vol. 115.

[bib38] Dolleman-Van Der Weel MJ, Witter MP (1996). Projections from the nucleus reuniens thalami to the entorhinal cortex, hippocampal field CA1, and the subiculum in the rat arise from different populations of neurons. The Journal of Comparative Neurology.

[bib39] Dolleman-Van der Weel MJ, Lopes da Silva FH, Witter MP (1997). Nucleus reuniens thalami modulates activity in hippocampal field CA1 through excitatory and inhibitory mechanisms. The Journal of Neuroscience.

[bib40] Eichenbaum H, Kuperstein M, Fagan A, Nagode J (1987). Cue-sampling and goal-approach correlates of hippocampal unit activity in rats performing an odor-discrimination task. The Journal of Neuroscience.

[bib41] Eichenbaum H (2017). Prefrontal-hippocampal interactions in episodic memory. Nature Reviews. Neuroscience.

[bib42] Farooq U, Dragoi G (2019). Emergence of preconfigured and plastic time-compressed sequences in early postnatal development. Science.

[bib43] Feng T, Silva D, Foster DJ (2015). Dissociation between the experience-dependent development of hippocampal theta sequences and single-trial phase precession. The Journal of Neuroscience.

[bib44] Ferbinteanu J, Shapiro ML (2003). Prospective and retrospective memory coding in the hippocampus. Neuron.

[bib45] Ferraris M, Ghestem A, Vicente AF, Nallet-Khosrofian L, Bernard C, Quilichini PP (2018). The Nucleus Reuniens Controls Long-Range Hippocampo-Prefrontal Gamma Synchronization during Slow Oscillations. The Journal of Neuroscience.

[bib46] Fletcher PC, Henson RN (2001). Frontal lobes and human memory: insights from functional neuroimaging. Brain.

[bib47] Foster DJ (2017). Replay Comes of Age. Annual Review of Neuroscience.

[bib48] Frankland PW, Bontempi B, Talton LE, Kaczmarek L, Silva AJ (2004). The involvement of the anterior cingulate cortex in remote contextual fear memory. Science.

[bib49] Freund TF, Antal M (1988). GABA-containing neurons in the septum control inhibitory interneurons in the hippocampus. Nature.

[bib50] Fried I, MacDonald KA, Wilson CL (1997). Single neuron activity in human hippocampus and amygdala during recognition of faces and objects. Neuron.

[bib51] Frotscher M, Léránth C (1985). Cholinergic innervation of the rat hippocampus as revealed by choline acetyltransferase immunocytochemistry: a combined light and electron microscopic study. The Journal of Comparative Neurology.

[bib52] Fuhrmann F, Justus D, Sosulina L, Kaneko H, Beutel T, Friedrichs D, Schoch S, Schwarz MK, Fuhrmann M, Remy S (2015). Locomotion, Theta Oscillations, and the Speed-Correlated Firing of Hippocampal Neurons Are Controlled by a Medial Septal Glutamatergic Circuit. Neuron.

[bib53] Gonzalez-Sulser A, Parthier D, Candela A, McClure C, Pastoll H, Garden D, Sürmeli G, Nolan MF (2014). GABAergic projections from the medial septum selectively inhibit interneurons in the medial entorhinal cortex. The Journal of Neuroscience.

[bib54] Green JD, Arduini AA (1954). Hippocampal electrical activity in arousal. Journal of Neurophysiology.

[bib55] Griffith WH, Matthews RT (1986). Electrophysiology of AChE-positive neurons in basal forebrain slices. Neuroscience Letters.

[bib56] Gulyás AI, Seress L, Tóth K, Acsády L, Antal M, Freund TF (1991). Septal GABAergic neurons innervate inhibitory interneurons in the hippocampus of the macaque monkey. Neuroscience.

[bib57] Guo KH, Li DP, Gu HY, Jie X, Yao ZB (2014). Postnatal development of nestin positive neurons in rat basal forebrain: different onset and topography with choline acetyltransferase and parvalbumin expression. International Journal of Developmental Neuroscience.

[bib58] Gupta AS, van der Meer MA, Touretzky DS, Redish AD (2012). Segmentation of spatial experience by hippocampal theta sequences. Nature Neuroscience.

[bib59] Hafting T, Fyhn M, Molden S, Moser MB, Moser EI (2005). Microstructure of a spatial map in the entorhinal cortex. Nature.

[bib60] Hallock HL, Wang A, Griffin AL (2016). Ventral Midline Thalamus Is Critical for Hippocampal-Prefrontal Synchrony and Spatial Working Memory. The Journal of Neuroscience.

[bib61] Hangya B, Borhegyi Z, Szilágyi N, Freund TF, Varga V (2009). GABAergic neurons of the medial septum lead the hippocampal network during theta activity. The Journal of Neuroscience.

[bib62] Hassabis D, Kumaran D, Vann SD, Maguire EA (2007). Patients with hippocampal amnesia cannot imagine new experiences. PNAS.

[bib63] Hembrook JR, Onos KD, Mair RG (2012). Inactivation of ventral midline thalamus produces selective spatial delayed conditional discrimination impairment in the rat. Hippocampus.

[bib64] Henderson Z, Morris NP, Grimwood P, Fiddler G, Yang HW, Appenteng K (2001). Morphology of local axon collaterals of electrophysiologically characterised neurons in the rat medial septal/ diagonal band complex. The Journal of Comparative Neurology.

[bib65] Henderson Z, Lu CB, Janzsó G, Matto N, McKinley CE, Yanagawa Y, Halasy K (2010). Distribution and role of Kv3.1b in neurons in the medial septum diagonal band complex. Neuroscience.

[bib66] Hoover WB, Vertes RP (2007). Anatomical analysis of afferent projections to the medial prefrontal cortex in the rat. Brain Structure & Function.

[bib67] Hyman JM, Zilli EA, Paley AM, Hasselmo ME (2005). Medial prefrontal cortex cells show dynamic modulation with the hippocampal theta rhythm dependent on behavior. Hippocampus.

[bib68] Hyman JM, Zilli EA, Paley AM, Hasselmo ME (2010). Working Memory Performance Correlates with Prefrontal-Hippocampal Theta Interactions but not with Prefrontal Neuron Firing Rates. Frontiers in Integrative Neuroscience.

[bib69] Ito HT, Zhang SJ, Witter MP, Moser EI, Moser MB (2015). A prefrontal-thalamo-hippocampal circuit for goal-directed spatial navigation. Nature.

[bib70] Jankowski MM, Islam MN, Wright NF, Vann SD, Erichsen JT, Aggleton JP, O’Mara SM (2014). Nucleus reuniens of the thalamus contains head direction cells. eLife.

[bib71] Jay TM, Glowinski J, Thierry AM (1989). Selectivity of the hippocampal projection to the prelimbic area of the prefrontal cortex in the rat. Brain Research.

[bib72] Jeffery KJ, Donnett JG, O’Keefe J (1995). Medial septal control of theta-correlated unit firing in the entorhinal cortex of awake rats. Neuroreport.

[bib73] Jimenez JC, Su K, Goldberg AR, Luna VM, Biane JS, Ordek G, Zhou P, Ong SK, Wright MA, Zweifel L (2018). Anxiety Cells in a Hippocampal-Hypothalamic Circuit. Neuron.

[bib74] Johnson A, Redish AD (2007). Neural ensembles in CA3 transiently encode paths forward of the animal at a decision point. The Journal of Neuroscience.

[bib75] Joo HR, Frank LM (2018). The hippocampal sharp wave-ripple in memory retrieval for immediate use and consolidation. Nature Reviews. Neuroscience.

[bib76] Jung MW, Wiener SI, McNaughton BL (1994). Comparison of spatial firing characteristics of units in dorsal and ventral hippocampus of the rat. The Journal of Neuroscience.

[bib77] Justus D, Dalügge D, Bothe S, Fuhrmann F, Hannes C, Kaneko H, Friedrichs D, Sosulina L, Schwarz I, Elliott DA, Schoch S, Bradke F, Schwarz MK, Remy S (2017). Glutamatergic synaptic integration of locomotion speed via septoentorhinal projections. Nature Neuroscience.

[bib78] Kay K, Chung JE, Sosa M, Schor JS, Karlsson MP, Larkin MC, Liu DF, Frank LM (2020). Constant Sub-second Cycling between Representations of Possible Futures in the Hippocampus. Cell.

[bib79] Ketz NA, Jensen O, O’Reilly RC (2015). Thalamic pathways underlying prefrontal cortex-medial temporal lobe oscillatory interactions. Trends in Neurosciences.

[bib80] Kimura H, McGeer PL, Peng F, McGeer EG (1980). Choline acetyltransferase-containing neurons in rodent brain demonstrated by immunohistochemistry. Science.

[bib81] King C, Recce M, O’Keefe J (1998). The rhythmicity of cells of the medial septum/diagonal band of Broca in the awake freely moving rat: relationships with behaviour and hippocampal theta. The European Journal of Neuroscience.

[bib82] Kinnavane L, Amin E, Olarte-Sanchez CM, Aggleton JP (2016). Detecting and discriminating novel objects: The impact of perirhinal cortex disconnection on hippocampal activity patterns. Hippocampus.

[bib83] Kiss J, Borhegyi Z, Csaky A, Szeiffert G, Leranth C (1997). Parvalbumin-containing cells of the angular portion of the vertical limb terminate on calbindin-immunoreactive neurons located at the border between the lateral and medial septum of the rat. Experimental Brain Research.

[bib84] Kjelstrup KG, Tuvnes FA, Steffenach HA, Murison R, Moser EI, Moser MB (2002). Reduced fear expression after lesions of the ventral hippocampus. PNAS.

[bib85] Kjelstrup KB, Solstad T, Brun VH, Hafting T, Leutgeb S, Witter MP, Moser EI, Moser MB (2008). Finite scale of spatial representation in the hippocampus. Science.

[bib86] Koenig J, Linder AN, Leutgeb JK, Leutgeb S (2011). The spatial periodicity of grid cells is not sustained during reduced theta oscillations. Science.

[bib87] Kohler C, Chan-Palay V, Wu JY (1984). Septal neurons containing glutamic acid decarboxylase immunoreactivity project to the hippocampal region in the rat brain. Anatomy and Embryology.

[bib88] Komorowski RW, Garcia CG, Wilson A, Hattori S, Howard MW, Eichenbaum H (2013). Ventral hippocampal neurons are shaped by experience to represent behaviorally relevant contexts. The Journal of Neuroscience.

[bib89] Langston RF, Ainge JA, Couey JJ, Canto CB, Bjerknes TL, Witter MP, Moser EI, Moser MB (2010). Development of the spatial representation system in the rat. Science.

[bib90] Lara-Vasquez A, Espinosa N, Duran E, Stockle M, Fuentealba P (2016). Midline thalamic neurons are differentially engaged during hippocampus network oscillations. Scientific Reports.

[bib91] Lavenex P, Suzuki WA, Amaral DG (2002). Perirhinal and parahippocampal cortices of the macaque monkey: projections to the neocortex. The Journal of Comparative Neurology.

[bib92] Layfield DM, Patel M, Hallock H, Griffin AL (2015). Inactivation of the nucleus reuniens/rhomboid causes a delay-dependent impairment of spatial working memory. Neurobiology of Learning and Memory.

[bib93] Lee MG, Chrobak JJ, Sik A, Wiley RG, Buzsaki G (1994). Hippocampal theta activity following selective lesion of the septal cholinergic system. Neuroscience.

[bib94] Lee AK, Wilson MA (2002). Memory of sequential experience in the hippocampus during slow wave sleep. Neuron.

[bib95] Malik R, Li Y, Schamiloglu S, Sohal V (2021). Top-down Control of Hippocampal Signal-to-Noise by Prefrontal Long-Range Inhibition. bioRxiv.

[bib96] Manseau F, Danik M, Williams S (2005). A functional glutamatergic neurone network in the medial septum and diagonal band area. The Journal of Physiology.

[bib97] Markram H, Segal M (1990). Electrophysiological characteristics of cholinergic and non-cholinergic neurons in the rat medial septum-diagonal band complex. Brain Research.

[bib98] Maviel T, Durkin TP, Menzaghi F, Bontempi B (2004). Sites of neocortical reorganization critical for remote spatial memory. Science.

[bib99] McKenna JT, Vertes RP (2004). Afferent projections to nucleus reuniens of the thalamus. The Journal of Comparative Neurology.

[bib100] McNaughton N, Ruan M, Woodnorth MA (2006). Restoring theta-like rhythmicity in rats restores initial learning in the Morris water maze. Hippocampus.

[bib101] Meyer AF, O’Keefe J, Poort J (2020). Two Distinct Types of Eye-Head Coupling in Freely Moving Mice. Current Biology.

[bib102] Miller VM, Best PJ (1980). Spatial correlates of hippocampal unit activity are altered by lesions of the fornix and endorhinal cortex. Brain Research.

[bib103] Mitchell SJ, Rawlins JN, Steward O, Olton DS (1982). Medial septal area lesions disrupt theta rhythm and cholinergic staining in medial entorhinal cortex and produce impaired radial arm maze behavior in rats. The Journal of Neuroscience.

[bib104] Miyashita Y (2004). Cognitive memory: cellular and network machineries and their top-down control. Science.

[bib105] Mizumori SJ, Perez GM, Alvarado MC, Barnes CA, McNaughton BL (1990). Reversible inactivation of the medial septum differentially affects two forms of learning in rats. Brain Research.

[bib106] Moser EI, Moser MB (2013). Grid cells and neural coding in high-end cortices. Neuron.

[bib107] Muenzinger KF (1956). On the origin and early use of the term vicarious trial and error (VTE. Psychological Bulletin.

[bib108] O’Keefe J, Nadel L (1978). The Hippocampus as a Cognitive Map.

[bib109] O’Keefe J, Recce ML (1993). Phase relationship between hippocampal place units and the EEG theta rhythm. Hippocampus.

[bib110] O’Neill PK, Gordon JA, Sigurdsson T (2013). Theta oscillations in the medial prefrontal cortex are modulated by spatial working memory and synchronize with the hippocampus through its ventral subregion. The Journal of Neuroscience.

[bib111] Panula P, Emson P, Wu JY (1980). Demonstration of enkephalin-, substance p- and glutamate decarboxylase-like immunoreactivity in cultured cells derived from newborn rat neostriatum. Histochemistry.

[bib112] Petersen PC, Buzsaki G (2020). Cooling of Medial Septum Reveals Theta Phase Lag Coordination of Hippocampal Cell Assemblies. Neuron.

[bib113] Pfeiffer BE, Foster DJ (2013). Hippocampal place-cell sequences depict future paths to remembered goals. Nature.

[bib114] Phillips ML, Robinson HA, Pozzo-Miller L (2019). Ventral hippocampal projections to the medial prefrontal cortex regulate social memory. eLife.

[bib115] Posner MI, Rothbart MK, Sheese BE, Tang Y (2007). The anterior cingulate gyrus and the mechanism of self-regulation. Cognitive, Affective & Behavioral Neuroscience.

[bib116] Prasad JA, Chudasama Y (2013). Viral tracing identifies parallel disynaptic pathways to the hippocampus. The Journal of Neuroscience.

[bib117] Qasim SE, Fried I, Jacobs J (2021). Phase precession in the human hippocampus and entorhinal cortex. Cell.

[bib118] Rajasethupathy P, Sankaran S, Marshel JH, Kim CK, Ferenczi E, Lee SY, Berndt A, Ramakrishnan C, Jaffe A, Lo M (2015). Projections from neocortex mediate top-down control of memory retrieval. Nature.

[bib119] Ramanathan KR, Ressler RL, Jin J, Maren S (2018). Nucleus Reuniens Is Required for Encoding and Retrieving Precise, Hippocampal-Dependent Contextual Fear Memories in Rats. The Journal of Neuroscience.

[bib120] Ranganath C, Cohen MX, Dam C, D’Esposito M (2004). Inferior temporal, prefrontal, and hippocampal contributions to visual working memory maintenance and associative memory retrieval. The Journal of Neuroscience.

[bib121] Redish AD (2016). Vicarious trial and error. Nature Reviews. Neuroscience.

[bib122] Robinson J, Manseau F, Ducharme G, Amilhon B, Vigneault E, El Mestikawy S, Williams S (2016). Optogenetic Activation of Septal Glutamatergic Neurons Drive Hippocampal Theta Rhythms. The Journal of Neuroscience.

[bib123] Rolls ET, Stringer SM, Elliot T (2006). Entorhinal cortex grid cells can map to hippocampal place cells by competitive learning. Network.

[bib124] Rozeske RR, Jercog D, Karalis N, Chaudun F, Khoder S, Girard D, Winke N, Herry C (2018). Prefrontal-Periaqueductal Gray-Projecting Neurons Mediate Context Fear Discrimination. Neuron.

[bib125] Rushworth MF, Noonan MP, Boorman ED, Walton ME, Behrens TE (2011). Frontal cortex and reward-guided learning and decision-making. Neuron.

[bib126] Sakurai Y (2002). Coding of auditory temporal and pitch information by hippocampal individual cells and cell assemblies in the rat. Neuroscience.

[bib127] Sargolini F, Fyhn M, Hafting T, McNaughton BL, Witter MP, Moser MB, Moser EI (2006). Conjunctive representation of position, direction, and velocity in entorhinal cortex. Science.

[bib128] Savelli F, Knierim JJ (2010). Hebbian analysis of the transformation of medial entorhinal grid-cell inputs to hippocampal place fields. Journal of Neurophysiology.

[bib129] Schacter DL, Addis DR, Buckner RL (2008). Episodic simulation of future events: concepts, data, and applications. Annals of the New York Academy of Sciences.

[bib130] Schiller D, Eichenbaum H, Buffalo EA, Davachi L, Foster DJ, Leutgeb S, Ranganath C (2015). Memory and Space: Towards an Understanding of the Cognitive Map. The Journal of Neuroscience.

[bib131] Schlesiger MI, Cannova CC, Boublil BL, Hales JB, Mankin EA, Brandon MP, Leutgeb JK, Leibold C, Leutgeb S (2015). The medial entorhinal cortex is necessary for temporal organization of hippocampal neuronal activity. Nature Neuroscience.

[bib132] Schlesiger MI, Boublil BL, Hales JB, Leutgeb JK, Leutgeb S (2018). Hippocampal Global Remapping Can Occur without Input from the Medial Entorhinal Cortex. Cell Reports.

[bib133] Scoville WB, Milner B (1957). Loss of recent memory after bilateral hippocampal lesions. J Neurol Neurosurg Psychiatry.

[bib134] Shin JD, Tang W, Jadhav SP (2019). Dynamics of Awake Hippocampal-Prefrontal Replay for Spatial Learning and Memory-Guided Decision Making. Neuron.

[bib135] Shute CC, Lewis PR (1967). The ascending cholinergic reticular system: neocortical, olfactory and subcortical projections. Brain : A Journal of Neurology.

[bib136] Si B, Treves A (2009). The role of competitive learning in the generation of DG fields from EC inputs. Cognitive Neurodynamics.

[bib137] Siapas AG, Lubenov EV, Wilson MA (2005). Prefrontal phase locking to hippocampal theta oscillations. Neuron.

[bib138] Skaggs WE, McNaughton BL, Wilson MA, Barnes CA (1996). Theta phase precession in hippocampal neuronal populations and the compression of temporal sequences. Hippocampus.

[bib139] Solstad T, Boccara CN, Kropff E, Moser MB, Moser EI (2008). Representation of geometric borders in the entorhinal cortex. Science.

[bib140] Sotty F, Danik M, Manseau F, Laplante F, Quirion R, Williams S (2003). Distinct electrophysiological properties of glutamatergic, cholinergic and GABAergic rat septohippocampal neurons: novel implications for hippocampal rhythmicity. The Journal of Physiology.

[bib141] Stumpf C, Petsche H, Gogolak G (1962). The significance of the rabbit’s septum as a relay station between the midbrain and the hippocampus. II. The differential influence of drugs upon both the septal cell firing pattern and the hippocampus theta activity. Electroencephalography and Clinical Neurophysiology.

[bib142] Svoboda E, McKinnon MC, Levine B (2006). The functional neuroanatomy of autobiographical memory: a meta-analysis. Neuropsychologia.

[bib143] Swanson LW, Kohler C (1986). Anatomical evidence for direct projections from the entorhinal area to the entire cortical mantle in the rat. The Journal of Neuroscience.

[bib144] Tang W, Shin JD, Jadhav SP (2021). Multiple time-scales of decision making in the hippocampus and prefrontal cortex. eLife.

[bib145] Tavares RM, Mendelsohn A, Grossman Y, Williams CH, Shapiro M, Trope Y, Schiller D (2015). A Map for Social Navigation in the Human Brain. Neuron.

[bib146] Tolman EC (1948). Cognitive maps in rats and men. Psychological Review.

[bib147] Tronel S, Sara SJ (2003). Blockade of NMDA receptors in prelimbic cortex induces an enduring amnesia for odor-reward associative learning. The Journal of Neuroscience.

[bib148] Van Cauter T, Poucet B, Save E (2008). Unstable CA1 place cell representation in rats with entorhinal cortex lesions. The European Journal of Neuroscience.

[bib149] Vandecasteele M, Varga V, Berenyi A, Papp E, Bartho P, Venance L, Freund TF, Buzsaki G (2014). Optogenetic activation of septal cholinergic neurons suppresses sharp wave ripples and enhances theta oscillations in the hippocampus. PNAS.

[bib150] Varga V, Hangya B, Kránitz K, Ludányi A, Zemankovics R, Katona I, Shigemoto R, Freund TF, Borhegyi Z (2008). The presence of pacemaker HCN channels identifies theta rhythmic GABAergic neurons in the medial septum. The Journal of Physiology.

[bib151] Vargha-Khadem F, Gadian DG, Watkins KE, Connelly A, Van Paesschen W, Mishkin M (1997). Differential effects of early hippocampal pathology on episodic and semantic memory. Science.

[bib152] Venditto SJC, Le B, Newman EL (2019). Place cell assemblies remain intact, despite reduced phase precession, after cholinergic disruption. Hippocampus.

[bib153] Vertes RP, Kocsis B (1997). Brainstem-diencephalo-septohippocampal systems controlling the theta rhythm of the hippocampus. Neuroscience.

[bib154] Vertes RP (2002). Analysis of projections from the medial prefrontal cortex to the thalamus in the rat, with emphasis on nucleus reuniens. The Journal of Comparative Neurology.

[bib155] Vertes RP (2004). Differential projections of the infralimbic and prelimbic cortex in the rat. Synapse.

[bib156] Vertes RP (2006). Interactions among the medial prefrontal cortex, hippocampus and midline thalamus in emotional and cognitive processing in the rat. Neuroscience.

[bib157] Vertes RP, Hoover WB, Szigeti-Buck K, Leranth C (2007). Nucleus reuniens of the midline thalamus: link between the medial prefrontal cortex and the hippocampus. Brain Research Bulletin.

[bib158] Wang Y, Romani S, Lustig B, Leonardo A, Pastalkova E (2015). Theta sequences are essential for internally generated hippocampal firing fields. Nature Neuroscience.

[bib159] Wang M, Foster DJ, Pfeiffer BE (2020). Alternating sequences of future and past behavior encoded within hippocampal theta oscillations. Science.

[bib160] Widmer H, Ferrigan L, Davies CH, Cobb SR (2006). Evoked slow muscarinic acetylcholinergic synaptic potentials in rat hippocampal interneurons. Hippocampus.

[bib161] Wills TJ, Cacucci F, Burgess N, O’Keefe J (2010). Development of the hippocampal cognitive map in preweanling rats. Science.

[bib162] Winson J (1974). Patterns of hippocampal theta rhythm in the freely moving rat. Electroencephalography and Clinical Neurophysiology.

[bib163] Winson J (1978). Loss of hippocampal theta rhythm results in spatial memory deficit in the rat. Science.

[bib164] Wood ER, Dudchenko PA, Robitsek RJ, Eichenbaum H (2000). Hippocampal neurons encode information about different types of memory episodes occurring in the same location. Neuron.

[bib165] Wouterlood FG, Saldana E, Witter MP (1990). Projection from the nucleus reuniens thalami to the hippocampal region: light and electron microscopic tracing study in the rat with the anterograde tracer Phaseolus vulgaris-leucoagglutinin. The Journal of Comparative Neurology.

[bib166] Xu W, Sudhof TC (2013). A neural circuit for memory specificity and generalization. Science.

[bib167] Ye X, Kapeller-Libermann D, Travaglia A, Inda MC, Alberini CM (2017). Direct dorsal hippocampal-prelimbic cortex connections strengthen fear memories. Nature Neuroscience.

[bib168] Zutshi I, Brandon MP, Fu ML, Donegan ML, Leutgeb JK, Leutgeb S (2018). Hippocampal Neural Circuits Respond to Optogenetic Pacing of Theta Frequencies by Generating Accelerated Oscillation Frequencies. Current Biology.

